# The origins of breast cancer associated with mammographic density: a testable biological hypothesis

**DOI:** 10.1186/s13058-018-0941-y

**Published:** 2018-03-07

**Authors:** Norman Boyd, Hal Berman, Jie Zhu, Lisa J. Martin, Martin J. Yaffe, Sofia Chavez, Greg Stanisz, Greg Hislop, Anna M. Chiarelli, Salomon Minkin, Andrew D. Paterson

**Affiliations:** 1 Princess Margaret Cancer Centre, 610 University Avenue, Room 9-502, Toronto, ON M5G 2M9 Canada; 2 Genetics and Genome Biology, Hospital for Sick Children Research Institute, Toronto, ON Canada; 3 Divisions of Epidemiology and Biostatistics, Dalla Lana School of Public Health, University of Toronto, Toronto, ON Canada; 4 Imaging Research, Sunnybrook Health Sciences Centre, Toronto, ON Canada; 5 Cancer Care Ontario, Toronto, ON Canada; 6 BC Cancer Agency, Vancouver, BC Canada

**Keywords:** Mammographic density, Two-stage model, Breast cancer, Carcinogenesis

## Abstract

**Background:**

Our purpose is to develop a testable biological hypothesis to explain the known increased risk of breast cancer associated with extensive percent mammographic density (PMD), and to reconcile the apparent paradox that although PMD decreases with increasing age, breast cancer incidence increases.

**Methods:**

We used the Moolgavkar model of carcinogenesis as a framework to examine the known biological properties of the breast tissue components associated with PMD that includes epithelium and stroma, in relation to the development of breast cancer. In this model, normal epithelial cells undergo a mutation to become intermediate cells, which, after further mutation, become malignant cells. A clone of such cells grows to become a tumor. The model also incorporates changes with age in the number of susceptible epithelial cells associated with menarche, parity, and menopause. We used measurements of the radiological properties of breast tissue in 4454 healthy subjects aged from 15 to 80+ years to estimate cumulative exposure to PMD (CBD) in the population, and we examined the association of CBD with the age-incidence curve of breast cancer in the population.

**Results:**

Extensive PMD is associated with a greater number of breast epithelial cells, lobules, and fibroblasts, and greater amounts of collagen and extracellular matrix. The known biological properties of these tissue components may, singly or in combination, promote the acquisition of mutations by breast epithelial cells specified by the Moolgavkar model, and the subsequent growth of a clone of malignant cells to form a tumor. We also show that estimated CBD in the population from ages 15 to 80+ years is closely associated with the age-incidence curve of breast cancer in the population.

**Conclusions:**

These findings are consistent with the hypothesis that the biological properties of the breast tissue components associated with PMD increase the probability of the transition of normal epithelium to malignant cells, and that the accumulation of mutations with CBD may influence the age-incidence curve of breast cancer. This hypothesis gives rise to several testable predictions.

**Electronic supplementary material:**

The online version of this article (10.1186/s13058-018-0941-y) contains supplementary material, which is available to authorized users.

## Background

Percent mammographic density (PMD) is one of the strongest known risk factors for breast cancer [1]. Fibroglandular tissue attenuates X-rays more than does fat [2], and it appears white (dense) in mammograms, whereas adipose tissue appears dark. PMD, illustrated in Additional file 1: Figure S1a, refers to the area of white tissue divided by the total area of the breast in the image. The dense area and PMD are both associated positively with risk of breast cancer, and PMD is the stronger risk factor [3]. The nondense area is associated inversely with risk of breast cancer [3, 4]. The increased risk of breast cancer associated with PMD persists for at least 8–10 years after the date of the mammogram used to assess PMD [5, 6], and it cannot be explained by the “masking” of cancers by dense breast tissue [6, 7]. In addition to an increased risk of breast cancer, PMD is also associated with an increased risk of lesions that are thought to be nonobligate precursors of breast cancer [8].

Average PMD in the population decreases with increasing age [5]. A cross-sectional study of 11,000 women in 22 countries showed that average PMD declined with increasing age. Decline was present before and after menopause and was most pronounced over the menopausal transition [9]. Longitudinal data within individuals has shown average reductions in PMD of from 5% [10] to 8% [11] respectively over 10 to 5 years.

Similar variations in breast tissue composition can be seen using measures of fat and water obtained by magnetic resonance (MR). Radiologically dense breast tissue and breast water both reflect fibroglandular breast tissue (Additional file 1: Figure S1b).

Antoni et al. showed in a meta-analysis of 19 studies with a total of > 24,000 breast cancer cases [12] that, relative to women in the lowest density category, women in the highest density category had 3.1-fold (95% CI 2.2–4.2) and 3.2-fold (1.7–5.9) increased risk of estrogen receptor-positive (ER+) and ER− breast cancer, respectively. In case-only analyses, relative risks of breast tumors for ER+ versus ER− were 1.13 (95% CI 0.89–1.42) for medium versus minimal mammographic density (MD). MD remained associated with screen-detected ER+ tumors. In eight contributing studies, the association of MD did not differ by HER2 status. Variations in the distribution by age of ER+ and ER− breast cancer are likely to be influenced by factors other than MD.

Breast cancer risk increases with increasing extent of PMD, and estimates of attributable risk (which assume causality) suggest that 30–50% of breast cancer may be attributed to the most extensive categories of PMD [5, 6]. Although MD is associated with relative and attributable risks that are large compared with other risk factors for the disease, the accuracy of risk prediction in individuals is modest [13].

The mechanisms that underlie the association of PMD with risk of breast cancer are not well defined [14], and the apparent paradox that with increasing age average PMD decreases while breast cancer incidence increases remains unexplained. We have previously proposed [15] that the radiological features of the breast of PMD provide an index of cumulative exposure to events that influence the incidence of breast cancer, similar to the concept of “breast tissue aging” proposed by Pike et al. [16]. However, to date, there is only one published study to support this suggestion [10].

In this paper, we develop a testable biological hypothesis to explain the origins of breast cancer associated with mammographic density. We summarize evidence that PMD reflects the relative quantities of epithelium, stroma, and fat in the breast, and we use a two-stage model of carcinogenesis as a framework to examine how the known biological properties of these tissues may influence the transition of normal breast epithelial cells to malignant cells [17]. We expect cumulative exposure to these biological factors to contribute to the age-specific incidence of breast cancer, and we examine the relationship between estimated cumulative exposure to PMD (CBD) in the population and the age-specific incidence of breast cancer.

## Methods

### Two-stage model of carcinogenesis in the breast

**Fig. 1:**
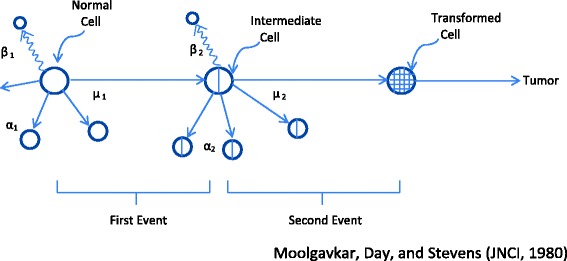
Two-stage model of carcinogenesis of Moolgavkar et al. [17]. In this model, normal stem cells, with a birth rate (α_1_) and rate of death (β_1_), can be transformed into cells of an intermediate form at a stochastic event rate (μ_1_) (the first mutation rate). These intermediate cells can divide into two further intermediate cells at a stochastic rate (α_2_), then die or differentiate at rate β_2_. In addition, intermediate cells can divide into one intermediate and one transformed (malignant) cell with a second stochastic event rate (μ_2_). The malignant cells are assumed to develop into a tumor after a deterministic lag time

Figure 1 shows the two-stage model described by Moolgavkar and colleagues applied to breast cancer [17]. Normal stem cells, with a birth rate (α_1_) divided into two daughter cells, and rate of death or differentiation (β_1_) can be transformed into cells of an intermediate form at a stochastic event rate (μ_1_) (the first mutation rate). These intermediate cells can divide into two further intermediate cells at a stochastic rate (α_2_) or die or differentiate at rate β_2_. In addition, intermediate cells can divide into one intermediate and one transformed (malignant) cell at a second stochastic event rate (μ_2_).

We recognize that molecular studies implicate several genetic changes in progression for breast cancer [18], and at the time of diagnosis, breast cancer cells contain multiple somatic mutations [19, 20]. In light of this, the two stages of the model may be viewed as rate-limiting steps in the process of breast carcinogenesis, with additional mutations occurring in intermediate cells that confer sustained proliferative and survival advantages to an expanding clone of cells that ultimately undergoes malignant transformation [19, 21]. We use the two-stage model solely as a framework for examining how the biological properties of the components of breast tissue associated with PMD might influence carcinogenesis in the breast.

The Moolgavkar model applied to female breast cancer is as follows:

$$ \mathrm{I}\left(\mathrm{t}\right)\approx {\upmu}_1{\upmu}_2\mathrm{F}\left(\mathrm{t}\right) $$

where *I*_t_ is breast cancer incidence at age *t*; μ_1_ and μ_2_ are the respective first and second mutation rates; and *F* is the susceptible cell population, modified by menarche, parity, and menopause, to age *t*. After assigning numerical values to μ_1_, μ_2_, and *F*, the model accurately predicts the age-specific incidence of breast cancer [17].

### Breast tissue associated with PMD

The histologic features of the breast associated with PMD have been examined using several approaches that include randomly selected breast tissue at forensic autopsy [22], as well as a comparison of radiologically dense and nondense regions in mastectomy specimens [23] and in surgical biopsies [24–26]. These approaches have shown similar results.

**Fig. 2:**
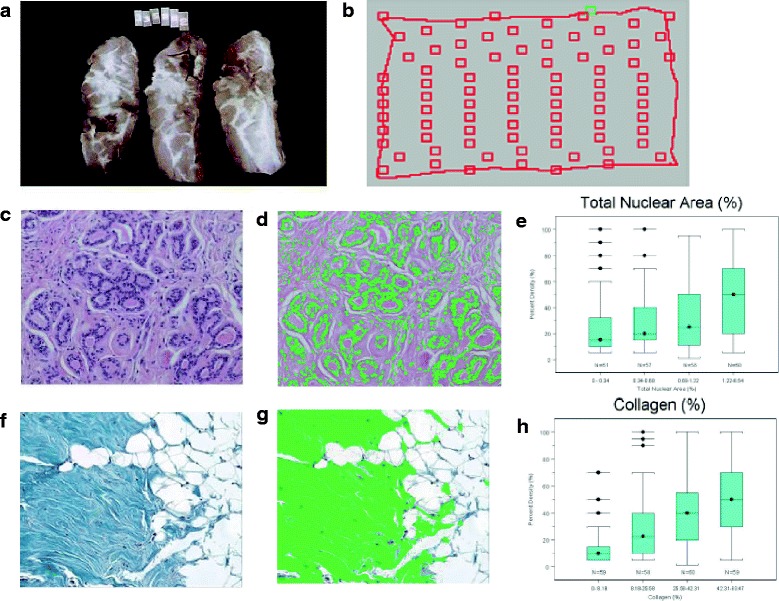
Breast tissue components associated with percent mammographic density (PMD). PMD was assessed in the BioVision (Faxitron Bioptics) image of the enucleated breast from which the section had been taken (**a**) [Li T, et al. Cancer Epidemiol Biomarkers Prev. 2005;14(2):343–9]. We used quantitative microscopy in randomly selected areas of the tissue section (**b**) to measure the total, epithelial, and nonepithelial nuclear areas (H&E stain in **c**) as an index of the number of cells (outlined in *green* in **d**), the area of collagen (H&E stain in **f** and Masson’s trichrome in **g**), and the glandular area. PMD was associated inversely with age, and, after age adjustment, positively with the nuclear area (**e**) of epithelial and nonepithelial cells, glandular area, and the area of collagen (**h**). Box plots in **e** and **h** show the associations of total nuclear area (**e**) and collagen (**h**) with PMD. The median values are shown as *horizontal lines*, and the *boxes* show the 25th and 75th percentiles of the distributions. Age, parity, and menopausal status were also associated with variations in one or more of these tissue components. Similar associations of PMD with these breast tissue components have been found in prophylactic mastectomies [13]. Original magnification ×10 (**c**, **d**, **f**, and **g**)

Selected results derived from randomly selected sections of breast tissue collected at forensic autopsy by Bartow et al. [27] are shown in Fig. 2. PMD was assessed in the BioVision (Faxitron Bioptics, Tucson, AZ, USA) image of the enucleated breast from which the section had been taken (Fig. 2a) [22]. We used quantitative microscopy in randomly selected areas of the tissue section (Fig. 2b) to measure the total, epithelial, and nonepithelial nuclear areas, which we used as an index of the number of cells (Fig. 2d), the area of Masson’s trichrome-stained collagen (Fig. 2g), and the glandular area. PMD was associated inversely with age, and after adjustment for age, positively with the nuclear area (a measure of the number of cells) (Fig. 2e) of epithelial and nonepithelial cells, glandular area, and the area of collagen (Fig. 2h). As shown in Additional file 1: Table S1, age, parity, and menopause were each associated inversely with one or more of these tissue components [22].

The stronger risk prediction seen with PMD compared with dense area suggests that the nondense area of the mammogram, which reflects fat, may provide some protection. A reduced risk of breast cancer associated with the nondense area of the mammogram has been shown in a meta-analysis by Pettersson et al. [3]. The mechanism underlying this protection is currently uncertain, but as shown in Fig. 2, breasts with low PMD are associated with fewer cells (as shown by nuclear area) and less extensive collagen, tissue components whose biological properties we show are associated with radiologically dense breast tissue and that may contribute to carcinogenesis in the breast. Further, aromatase activity in the breast is predominately in stromal preadipocytes [28] and is reduced when preadipocytes differentiate to mature adipocytes. The loss of this source of local estrogen production may contribute to the reduced risk of breast cancer associated with breast fat. As Fig. 2 shows, there can be wide variation in the number of cells (as shown by nuclear area) and the area of collagen between individuals, and it is not currently possible to assess these variations separately, or to link them to risk, using only currently available methods of imaging.

### Biological properties of breast tissue associated with PMD

#### Breast epithelium

Breast cancer is thought to originate in the epithelial cells of the terminal ductal lobular unit [29, 30] and to be the result of the accumulation of genetic mutations [21]. The greater number of epithelial cells and greater glandular area associated with PMD may be the result of either increased cell proliferation or a reduction in the rate of cell death. Both processes increase the size of the population of susceptible epithelial cells and increase the probability of a mutation. Some breast mitogens have been associated with risk of breast cancer [31–33], and proliferative activity in epithelial cells, as shown by the Ki-67 index, predicts risk of breast cancer in premenopausal women [34].

Although in adult life epithelial cells associated with PMD do not have an increased Ki-67 proliferative index [35], the greater number of epithelial cells associated with PMD in adult life may be the result of greater proliferation of progenitor cells during breast development, when susceptibility to carcinogens is also greatest [36]. The chemokine CCL2 has been detected in human mammary epithelium, and when overexpressed in mouse mammary epithelium, it induces a state of low-level inflammation that increases stromal density and risk of mammary cancer [37].

#### Stroma

Collagen, fibroblasts, other mesenchymal cells, and extracellular matrix (ECM) are stromal components that contribute to PMD. Selected biological properties of these components of stroma are discussed briefly in the following sections and summarized in Table 1. Some of the components of stroma considered here have multiple biological functions, and we have selected those functions that appear most likely to be relevant to the processes outlined above in the two-stage model of carcinogenesis.

**Table 1:** Selected biological properties of components of breast stroma

Tissue	Model	Biological effects	Two-stage model	References
Collagen	Transgenic mice (*Col1a1*) + PyVT transgene + MMTV promoter	2.5-fold increase in stromal collagen + 3-fold increase tumor incidence	1 + 2	[38]
	Human MEC + collagen gel	Increase migration of cancer stem cells.	2	[39, 40]
Fibroblasts	Human MEC + stromal cells in mouse	Genetically modified human stromal fibroblasts promote outgrowth of benign and malignant lesions from MEC.	1 + 2	[41]
	Human cells in culture + mouse models	TGF-β and HGF produced by stromal fibroblasts inhibit and stimulate, respectively, proliferation in adjacent epithelial cells.	1 + 2	[42–44]
		TGF-β promotes epithelial-mesenchymal transition and changes in the microenvironment that promote tumor progression.	2	[44, 45]
	Cancer associated fibroblasts (CAFs)	CAFs in stroma associated with breast cancer promote tumor dissemination.	2	[42, 46–49, 56]
Other cells	Aromatase in stromal preadipocytes in various models including human breast tissue	Estrogen produced by aromatase increases MEC proliferation + tumor growth.	1 + 2	[50–55]
ECM	Human breast tissue			
	Proteoglycans	Overexpression of lumican and decorin in PMD and breast cancer bind growth factors.	1 + 2	[59]
	MMP-3	Metalloproteinases regulate stromal matrix and the activation of growth factors.	1 + 2	[60, 61]
	Stiffness	Promotes tumorigenesis and growth		[57, 58]

*Abbreviations: ECM* Extracellular matrix, *MEC* Mammary epithelial cell, *PyVT* Polyomavirus middle T antigen, *MMTV* Mouse mammary tumor virus, *TGF-β* Transforming growth factor-β, *HGF* Hepatocyte growth factor, *MMP-3* Matrix metalloproteinase 3, *PMD*: Percent mammographic density

##### Collagen

Provenzano et al. [38] showed in a bitransgenic mouse tumor model, with both increased density of stromal collagen (*Col1a1*^tmJae^) and carrying the polyoma middle T transgene under the control of mammary-specific mouse mammary tumor virus promoter, that both epithelial cell proliferation and tumor formation were increased. Tumor formation increased approximately threefold, and tumors had a more invasive phenotype and a greater frequency of metastasis [38].

Preliminary human data suggest that periductal aligned collagen fibrils, rather than amorphous collagen, is associated with PMD [39]. Aligned collagen matrices also enhances the migration of cancer stem cells [40].

##### Fibroblasts

Stromal fibroblasts are the principal source of collagen and can regulate the morphogenesis of breast epithelial cells. Kuperwasser et al. showed that human stromal fibroblasts from reduction mammoplasty, immortalized with human telomerase, and implanted with normal human mammary epithelial cells (MECs) into the cleared mammary fat pad of severe combined immunodeficiency mice, resulted in the outgrowth of benign and malignant epithelial lesions [41].

Stromal fibroblasts regulate the growth of epithelial cells in part through the secretion of growth factors and chemokines [42] that include hepatocyte growth factor (HGF), insulin-like growth factor 1 (IGF-1), and transforming growth factor-β (TGF-β). HGF and IGF-1 both promote epithelial cell proliferation and tumor growth. Stromal fibroblast-derived TGF-β [43] inhibits MEC proliferation in vivo but can promote malignant behavior through diverse mechanisms that include stimulation of epithelial-mesenchymal transition [44]. TGF-β also has several effects on the microenvironment, including increasing ECM and inducing endothelial cell recruitment and proliferation, that promote tumor progression (reviewed in [45]). Fibroblasts also deposit ECM and produce collagen types I, III, and V and fibronectin (reviewed in [43]). Fibroblasts derived from disease-free breasts with extensive PMD promote adipocyte differentiation in culture and show decreased expression of CD36 [46] (*see below*).

The stroma associated with breast cancer contains fibroblasts (cancer-associated fibroblasts [CAFs]) that produce chemokines, growth factors, and ECM proteins, which are thought to contribute to the dissemination of malignant tumors [43, 47], and foci of fibrous tissue within invasive breast cancer are associated with an increased risk of disease recurrence [48, 49].

##### Other cells

Aromatase activity in the breast is a source of estrogen that may stimulate proliferation of epithelial cells and promote the growth of malignant clones [50–53]. Aromatase activity in adipose tissue is expressed primarily in stromal mesenchymal preadipocytes rather than in lipid-laden adipocytes and is greatest in breast tissue where the ratio of fibroblasts to adipocytes is greatest [54], and most aromatase activity in the breast is in radiologically dense regions [50, 51, 53, 55]. The role of immune cells in PMD has received little attention to date, but Huo et al. showed in prophylactic mastectomy samples that radiologically dense areas of the breast contained fewer CD26 activated macrophages and more vimentin^+^/CD45 immune cells than nondense regions in the same individuals [23, 56].

##### Extracellular matrix

The ECM is comprised of collagens, fibronectin, laminins, polysaccharides, and proteoglycans, and it influences the changes that occur in the breast during pregnancy, lactation, involution, and tumorigenesis (*see* [57, 58] for reviews). Expression of the proteoglycans lumican and decorin, assessed by semiquantitative scoring of immunohistochemistry, is increased in stromal tissue associated with breast cancer and, in the absence of invasive breast cancer, in women with extensive PMD. PMD is associated with lumican and decorin scores and with duct fibrosis and collagen, but not with the tissue or ductal lobular density [59]. Proteoglycans bind growth factors, contribute to the mechanical integrity of tissues, and influence the stiffness of breast tissue that promotes tumorigenesis, tumor growth, and the invasion of malignant tissue [57, 58].

Radiologically dense breast tissue also has greater amounts of the stromal matrix regulatory protein tissue inhibitor of metalloproteinase 3 [60] that regulates stromal matrix, the activation of growth factors, and influences susceptibility to breast cancer [61]. In addition, the transmembrane receptor *CD36* controls adipogenesis and deposition of the ECM. *CD36*-knockout mice show increased collagen and decreased fat in the mammary gland, and reduced expression of *CD36* has been found to be associated with greater PMD and tumor stroma in human breast tissue [46].

Radiologically dense human breast tissue obtained from mastectomy specimens has been shown to promote the growth and progression of human carcinoma in situ xenografts in immunodeficient mice [62]. The biological properties shown in Table 1 have all, with the exception of collagen density, been observed in human cells or tissues, but only three (proteoglycan expression, matrix metalloproteinase 3 [MMP-3], and CD36 expression) have been examined to date in relation to PMD.

##### Genetic variants associated with histologic features

Twin and sister studies have shown that more than 60% of the variation in PMD in the population can be explained by additive genetic effects [63, 64]. Genetic variants associated with PMD dense or nondense areas are likely to be associated, directly or indirectly, with one or more of the tissue components that are reflected by these mammographic features.

Genome-wide association studies (GWASs) have identified some of the genetic variants associated with PMD. Here we limit our attention to the nine regions, comprised of eight genes and one locus on chromosome 8, shown using a two-stage design to be reproducibly associated with PMD adjusted for age and body mass index. Eight of the nine loci are also associated with the risk of breast cancer [65, 66]. Single-nucleotide polymorphisms (SNPs) near or in *PRDM6* and *THEM184B* and the locus on chromosome 8 have been associated with PMD, and SNPs near *AREG*, *ESR1*, *ZNF365*, *LSP1*, *IGF1*, and *SGSM3/MKL1* have all been associated with the area of dense tissue in the mammogram*.* The locus on chromosome 8 has also been associated with nondense area.

Although it is recognized that proximity of SNPs to genes may not identify causal genes [67, 68], functions for genes near eight of these regions were found by searching under the gene names in PubMed, the National Center for Biotechnology Information database of Genotypes and Phenotypes (dbGaP) of genotype-phenotype associations, and the GWAS Catalog (http://www.ebi.ac.uk/gwas/) [69]. The known functions of these eight genes of potential relevance to the components of breast tissue that are associated with PMD include the following:

- ***AREG*** encodes amphiregulin that binds epidermal growth factor receptor, stimulates cell growth and survival, and plays a role in the development of the mammary gland [70]. Amphiregulin also promotes the growth of fibroblasts, the expression of collagen and other genes associated with the ECM, and interacts with *TGF-β* to stimulate fibroblast proliferation [71].
- ***ESR1*** encodes ER-α that mediates the physiological effects of estrogen [72]. Estrogen influences epithelial cell proliferation, and the secretion of the pituitary hormones growth hormone and prolactin that are breast mitogens [32, 73–75].
- ***IGF-1*** encodes IGF-1 [76] and has mitogenic and antiapoptotic effects on breast epithelial cells [77]. Serum levels of IGF-1 have been associated with breast cancer risk in meta-analysis [31] and with PMD in some but not all studies (reviewed in [1]).
  Greater adult height is associated with risk of breast cancer [78] and has been positively associated with percent breast water (which, like PMD, reflects fibroglandular tissue) in young women [75] and with PMD [79] in adult women. Variants near *MKL1*, *SGSM3*, and *IGF1* (in Japanese subjects) are associated with height [80, 81].
- ***MKL1*** is the human homologue of a murine gene (*Bsac*) that, when overexpressed in mice that are double-knockout for tumor necrosis factor (TNF)-associated factor, protects murine embryonic fibroblasts against cell death induced by TNF [82].
- ***LSP1*** [83] does not currently have any described function that is specific to breast tissue, apart from the observed associations with PMD and breast cancer.

## Results

### Application of two-stage model to association of PMD with breast cancer

**Fig. 3:**
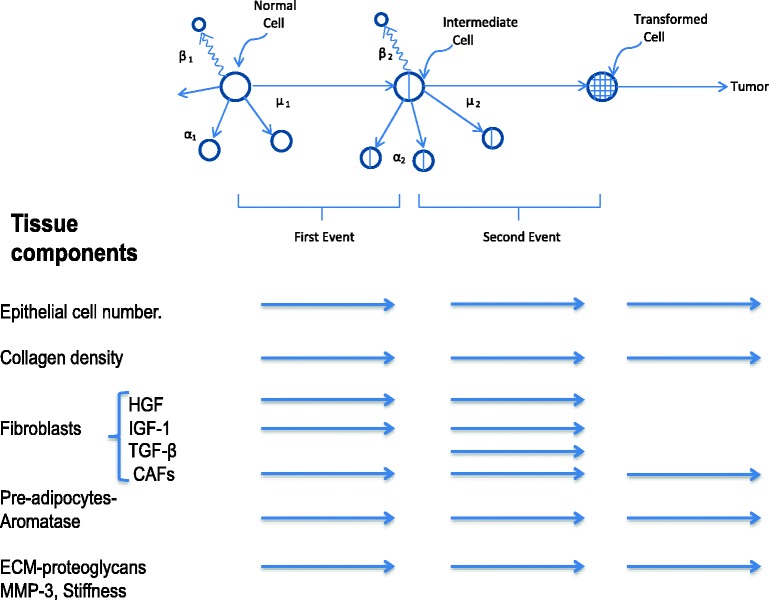
Proposed model of the two-stage model of carcinogenesis with risk of breast cancer in percent mammographic density. (The third column represents effects on the growth of a clone of malignant cells.) *CAF* Cancer-associated fibroblast, *ECM* Extracellular matrix, *HGF* Hepatocyte growth factor, *IGF-1* Insulin-like growth factor 1, *MMP-3* Matrix metalloproteinase 3, *TGF-β* Transforming growth factor-β

Figure 3 summarizes how the biological properties of the tissue components associated with PMD that are summarized in Table 1 might influence the first and second stages and transitions of the two-stage model. The third column represents the growth of a clone of malignant cells to become a detectable tumor.

The probability that the first event converts a normal epithelial stem cell to an intermediate cell is expected to be proportional to the number of cells at risk, their survival time, the number of cell divisions, and the dose and duration of exposure to mutagens [84]. As shown above, the extent of PMD is associated positively with the number of epithelial cells. Further, as shown in Table 1, experimental data show that epithelial cell proliferation and survival are increased by greater density of collagen, by the growth factors associated with fibroblasts and proteoglycans, by greater stiffness of the ECM, and by the local production of estrogen by aromatase, as well as by the influence of systemic hormones and growth factors.

These factors may operate singly or, more likely, in combination to promote the expansion of the pool of normal and intermediate cells, the acquisition of additional mutations that confer proliferative and survival advantages, the transition of intermediate cells to malignancy, and the subsequent growth of a clone of malignant cells to become a detectable tumor.

### Cumulative exposure to PMD and age-specific incidence of breast cancer

The postulated expansion with increasing age of the number of intermediate cells with mutations, together with continuing exposure to several components of the breast stroma that promote carcinogenesis, suggests that CBD may be related to the age-specific incidence of breast cancer [85, 86]. CBD may account for the observation that with increasing age, PMD and the total number of epithelial cells and lobular units decrease, whereas breast cancer incidence increases.

#### Estimated cumulative breast density in the population

We estimated CBD in the population using cross-sectional data from 4454 healthy females, predominately Caucasian and aged 15–81 years, who had participated in previous studies in which PMD was measured. Additional file 1: Table S2 shows selected characteristics of these subjects.

We measured PMD by mammography [87] (shown in Additional file 1: Figure S1a) in women over the age of 35 and by percent breast water by MR (shown in Additional file 1: Figure S1b) in those under 35 [75]. Both measures reflect fibroglandular breast tissue [88] and are strongly correlated with each other within the same individuals (*r*_s_ = 0.85) [75]. We used percent breast water by MR and PMD obtained in 100 adult women to calibrate MR measures in young women to the equivalent mammographic measure (*see* Table 2 footnote).

**Table 2:** Calculation of cumulative percent mammographic density

Age (years)	No. of subjects	Median calibrated percent water^a^	Median percent dense area	Years	Calibrated breast density years	Calibrated cumulative breast density years
15–19	974	51.7		5	258.5	258.6
20–24	86	46.5		5	232.5	491.1
25–29	83	47.0		5	235.0	726.1
30–34	15	46.3		5	231.5	957.6
35–39	49		45.9	5	229.6	1187.3
40–44	405		40.4	5	202.0	1389.3
45–49	654		37.3	5	186.6	1575.9
50–54	789		28.4	5	141.9	1717.8
55–59	546		23.8	5	119.0	1836.9
60–64	324		23.1	5	115.3	1952.2
65–69	259		19.2	5	96.1	2048.3
70–74	171		18.5	5	92.5	2140.8
75–79	76		18.3	5	91.3	2232.1
80+	23		15.2	5	76.0	2308.1

Percent breast water was calibrated to breast density equivalent as follows: calibrated percent breast density = (80.00813 − 1365.42571)/percent breast water

^a^In previous work, a random sample of 100 mothers was selected from among a total of 356 whose daughters had participated [75]. Their average age was 49.6 years (SD 4.2 years), and this and other characteristics were similar to those of mothers who did not have magnetic resonance imaging (*see* Table 1 in [75]). Mammographic measures of all mothers are included in the data shown in the table above

As shown in Table 2, we divided subjects into the same 5-year age categories in which breast cancer incidence in the population is reported. Median PMD decreased with increasing age and was 51.7% after calibration in the youngest group and 15.2% in the oldest. We multiplied the median PMD in each age category by the 5 years in the category to generate a variable we call “breast density years,” and we summed the product for each age group to give an estimate of CBD from ages 15 to 80+ years in the population.

#### Association of cumulative breast density with age-specific incidence of breast cancer

**Fig. 4:**
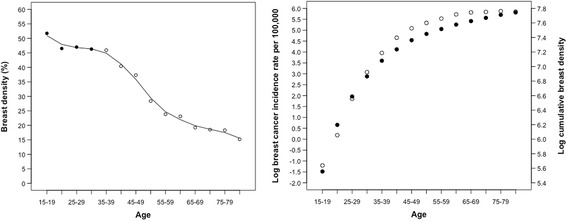
Cumulative breast density: observed and predicted breast cancer incidence. *Left*: Breast density according to age. Values derived from mammogram in *open circles*; values from calibrated measures derived from magnetic resonance in *closed circles*. *Right*: Log breast cancer incidence in *closed circles*, log cumulative breast density in *open circles*. Incidence data for age-specific incidence of invasive breast cancer for Canada were obtained from Curado MP et al. Cancer incidence in five continents. Vol. IX. IARC Scientific Publication no. 160. Lyon, France: IARC Press; 2007

We examine in Fig. 4 the association of CBD with the age-specific incidence of invasive breast cancer in Canada. We compared the log age-specific breast cancer incidence in the Canadian population predicted for each 5-year age group using regression models, one based on log age alone, one based on log CBD alone, and one based on log age + log CBD. We used *R*^2^ (the proportion of the total variance explained) and a comparison of the observed and predicted age-incidence curve of breast cancer to assess the fit of each model. The models, the associated coefficients, and the results are shown in Additional file 1: Table S3.

As shown in Fig. 4 and Additional file 1: Table S3, we found a strong association (*r*^2^ = 0.99) between log CBD and log breast cancer incidence in the population using the following model:

$$ \mathrm{Log}\ \mathrm{I}\left(\mathrm{t}\right)\approx \log\ {\left(\mathrm{CBD}\left(\mathrm{t}\right)\right)}^{\mathrm{k}} $$

where Log *I*_t_ is log breast cancer incidence at age *t*, CBD_t_ is the sum of median PMD in each age group (each multiplied by 5, the age interval) from age 15 to age *t*, and the exponent *k* has the estimated value of 3.5. The model based on log age alone was less strongly associated with breast cancer incidence (Additional file 1: Table S3), and the addition of log age to log CBD did not change the association with breast cancer incidence.

This model based on log CBD and the two-stage model of Moolgavkar et al. described above both accurately predict the age-specific incidence of breast cancer in the populations considered. The function *F*_t_ in the Moolgavkar model, and CBD_t_ in the present model both reflect variations in the number of susceptible cells in the breast modified by menarche, pregnancy, and menopause [89].

The strong correlation observed between log CBD and log breast cancer incidence cannot be explained by their shared association with age. Log CBD alone was a better predictor of age-specific log breast cancer incidence than was log age, and there was no change in prediction by a model containing both CBD and age.

## Discussion

The close relationship observed between log CBD and log breast cancer incidence is consistent with the hypothesis of carcinogenesis in which the accumulation of mutations or other molecular changes increases with increasing duration of exposure to PMD rather than with age. Limitations of our data include the cross-sectional observations and the ecological comparison with breast cancer incidence, as well as the small numbers at ages 30–34 and 80+ years. We estimated CBD from cross-sectional rather than longitudinal observations, using film rather than digital mammograms. However, longitudinal assessments of breast density in women aged 40 or older have shown a decline in average PMD with increasing age and menopause that is very similar to the differences seen here [10]. Further, Maskarinec et al. showed a strong association between cumulative density and age-specific breast cancer incidence in serial mammograms from 607 patients with breast cancer and 667 control subjects in the Hawaii component of the multiethnic cohort, in which the average age at first mammogram was 57 years [10]. However, the associations of cumulative density and breast cancer incidence with age were not examined [17].

CBD may also explain many of the known epidemiological associations with breast cancer risk. As shown above, the estimated size of the susceptible cell population of epithelial cells and epithelial cell proliferation are greatest at early ages and decline with increasing age. The greater amount of fibroglandular tissue, as shown by percent water, present at ages 15–18 may be related to the greater susceptibility of the breast at early ages to the effects of known exposures on risk of breast cancer, including radiation, alcohol, and smoking [36].

Early menarche is associated with an increased risk of breast cancer in later life [90] and advances the age at which fibroglandular breast tissue develops. This addition to the time of exposure will influence all estimates of PMD at later ages and will increase CBD. An early pregnancy and early menopause both reduce later risk of breast cancer and PMD [90]. The reductions in PMD associated with these events will influence all measures of PMD at later ages and reduce average CBD in parous and postmenopausal women, respectively. At least some of the effect of pregnancy in reducing risk of breast cancer has been shown to be mediated by the reduction in PMD associated with pregnancy [91].

Tamoxifen reduces PMD and risk of breast cancer, and reduction in PMD appears to predict response to adjuvant therapy with tamoxifen [92]. Progesterone as a postmenopausal replacement therapy has been shown to increase both PMD and breast cancer incidence, and the effect of progesterone on breast cancer incidence has been shown to be mediated through the effect on PMD [93]. The proliferation of mammary epithelium in response to progesterone is mediated by receptor activator of nuclear factor-κB ligand (RANKL), and increased expression of RANKL has been found to be associated with more extensive PMD in premenopausal women [94].

The biological hypothesis that we propose from the foregoing considerations is that the transition of breast epithelial cells from normal to malignant cells is completed more frequently in dense breast tissue than in nondense tissue. We propose that this transition is associated with the acquisition of mutations or other molecular changes in breast epithelial cells that increase in frequency with increasing exposure to both the amount and duration of PMD. We propose that the probability of acquiring mutations is influenced by the greater number of epithelial cells and by the several known biological properties of the stromal tissues that are associated with PMD, described in Table 1, by the amounts of such tissues, and by the duration of exposure to these influences. Proteoglycans and MMP-3 in the ECM of radiologically dense breast tissue have already been shown, in the absence of breast cancer, to be similar to those expressed in breast tissue associated with breast cancer.

Additional influences may include the greater number of stromal fibroblasts and associated chemokines associated with PMD that may, in the absence of breast cancer, resemble CAFs. CAFs can be distinguished from normal fibroblasts by markers and functional assays. Among these properties is the production of *TGF-β1*, which promotes epithelial-mesenchymal transition and has effects on the microenvironment that promote tumorigenesis and tumor invasion (reviewed in [45]). Epithelial-mesenchymal transition and other changes in the microenvironment may, in the absence of breast cancer, be more extensive in radiologically dense breast tissue than in nondense tissue [45].

## Conclusions

PMD has reproducibly been shown to be a strong risk factor for breast cancer that may account for a substantial fraction of the disease. The biological basis for this association is currently unknown, however. We have examined potential biological mechanisms for the risk of breast cancer associated with PMD using a two-stage model of carcinogenesis as a framework.

It is understood that it is the biological properties of the breast tissues associated with PMD, not the radiological properties, that are responsible for the association of PMD with risk of breast cancer. PMD is known to be associated with a greater number of epithelial cells, greater glandular area, a greater area of collagen, and a greater number of nonepithelial cells. The known biological properties of these breast tissue components increase the probability of mutation and of transition to malignant cells. The finding that CBD in healthy subjects in the population, estimated from cross-sectional observations in healthy women, was strongly associated with the age-specific incidence of breast cancer in Canada and is consistent with the accumulation of mutations with increasing time of exposure to CBD. This biological model gives rise to a number of testable predictions concerning the properties of breast tissue associated with PMD and suggests that the radiological features of the breast may be useful in the design, sampling, analysis, and interpretation of research on the biology of breast tissues in relation to breast cancer.

## Additional file

Additional file 1: **Figure S1. a** Examples of percent mammographic density (and grey scale). A = 0%; B = < 10%; C = 10 < 25%; D = 25% < 50%; E = 50% < 75%; F= > 75%. **b** Examples of percent breast water determined by magnetic resonance (and grayscale). Shows 0% (top left), 20% (top right), 60% (bottom left) and 90% (bottom right). **Table S1.** Associations of age, age at menarche, parity and menopausal status with breast tissue components. Values shown are regression coefficients, adjusted for age, and *p* values. **Table S2.** Selected characteristics of subjects according to study. **Table S3.** Comparison of observed and predicted age-specific breast cancer incidence using three predictive models (including young woman with calibrated percent water). (DOC 4356 kb)

## Abbreviations

CAF: Cancer-associated fibroblast
CBD: Cumulative exposure to breast density
ECM: Extracellular matrix
ER: Estrogen receptor
GWAS: Genome-wide association study
HGF: Hepatocyte growth factor
IGF-1: Insulin-like growth factor 1
MD: Mammographic density
MEC: Mammary epithelial cell
MMP-3: Matrix metalloproteinase 3
MMTV: Mouse mammary tumor virus
MR: Magnetic resonance
PMD: Percent mammographic density
PyVT: Polyomavirus middle T antigen
RANKL: Receptor activator of nuclear factor-κB ligand
SNP: Single-nucleotide polymorphism
TGF-β: Transforming growth factor-β
TNF: Tumor necrosis factor
