# Supplementary material for: The origins of breast cancer associated with mammographic density: a testable biological hypothesis
Source: Breast Cancer Res. 2018 Mar 7;20:17. doi: 10.1186/s13058-018-0941-y (PMC5842598; doi:10.1186/s13058-018-0941-y)
Supplement: Supplementary file 1 — Figure S1. a Examples of percent mammographic density (and grey scale). A = 0%; B = < 10%; C = 10 < 25%; D = 25% < 50%; E = 50% < 75%; F= > 75%. b Examples of percent breast water determined by magnetic resonance (and grayscale). Shows 0% (top left), 20% (top right), 60% (bottom left) and 90% (bottom right). Table S1. Associations of age, age at menarche, parity and menopausal status with breast tissue components. Values shown are regression coefficients, adjusted for age, and p values. Table S2. Selected characteristics of subjects according to study. Table S3. Comparison of observed and predicted age-specific breast cancer incidence using three predictive models (including young woman with calibrated percent water). (DOC 4356 kb) [file 13058_2018_941_MOESM1_ESM.doc]

**Supplementary Figure 1A**. **Examples of percent mammographic density (and grey scale)**. A=0%; B=<10%; C=10<25%; D=25%<50%; E=50%<75%; F=>75%.

**Supplementary Figure 1B. Examples of percent breast water determined by magnetic resonance (and grey scale).**

Shows 0% (top left), 20% (top right), 60% (bottom left) and 90% (bottom right)


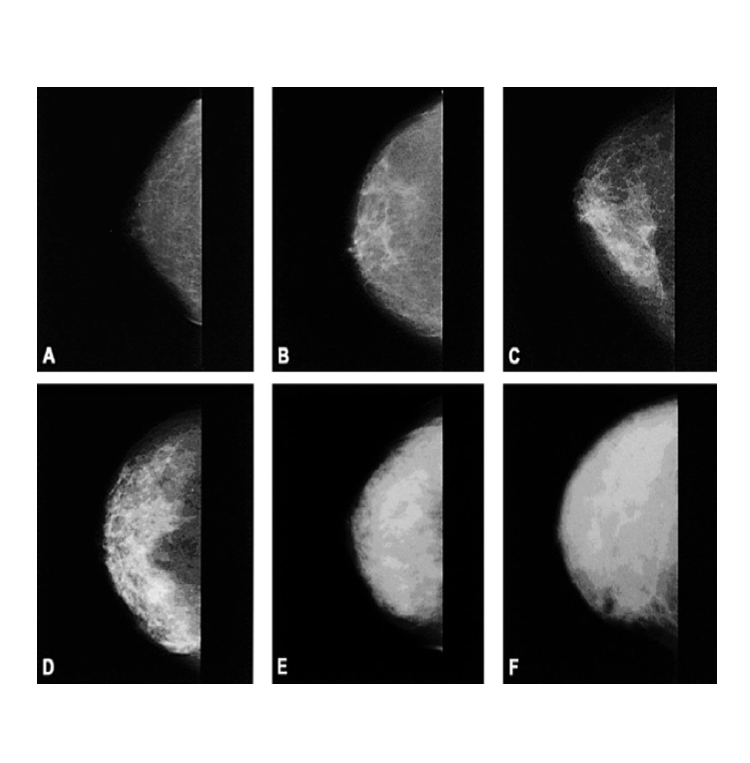


**Supplementary Figure 1A. b**Examples of variations in percent mammographic density (and grey scale): A=0%; B=0<10%; C=10<25%; D=25<50%; E=50<75%; F= ≥75%.

**Supplementary Figure 1B.** Examples of percent breast water determined by magnetic resonance (and grey scale).Shows 0% (top left), 20% (top right), 60% (bottom left) and 90% (bottom right)

100%

100%

50%

0%


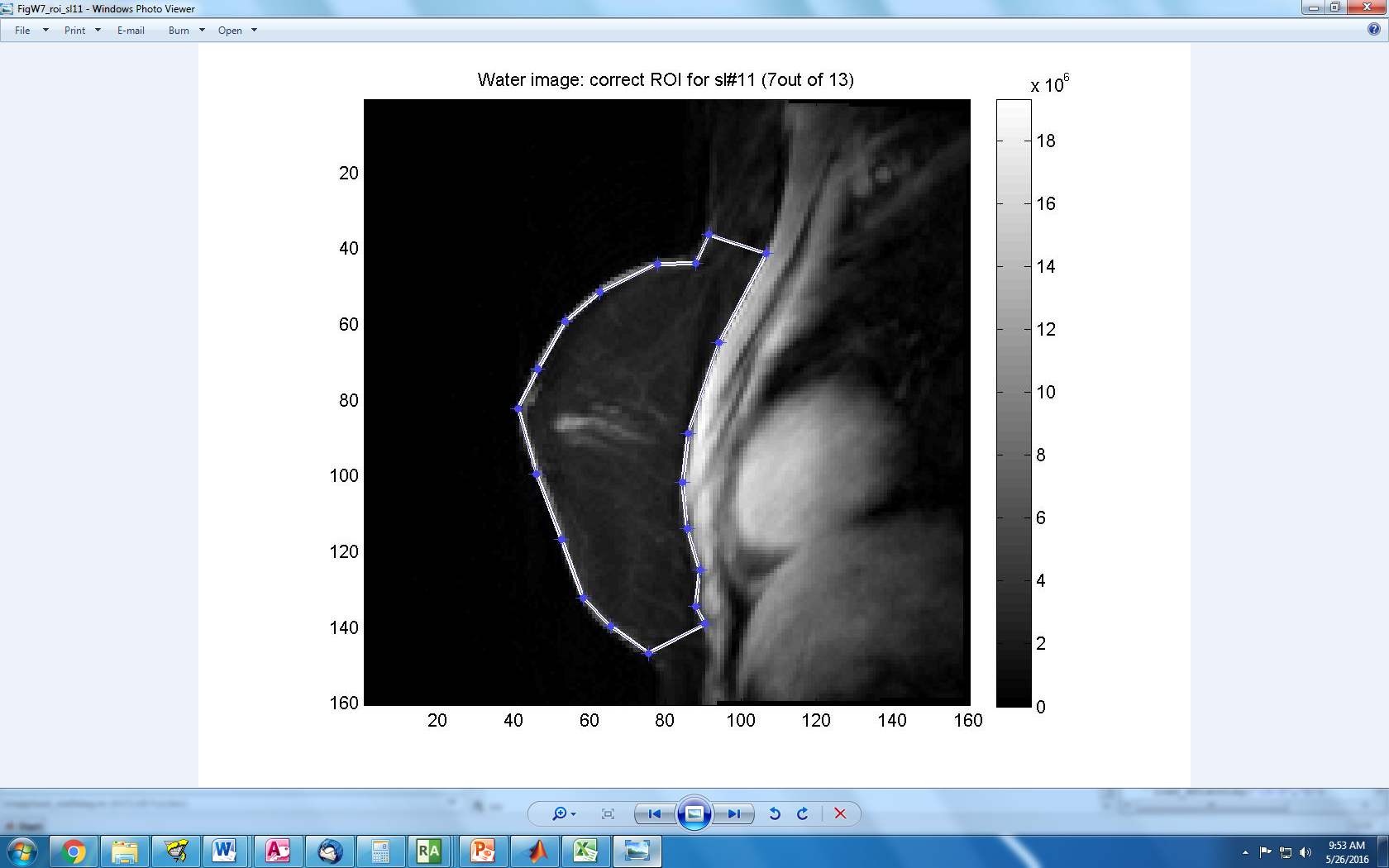

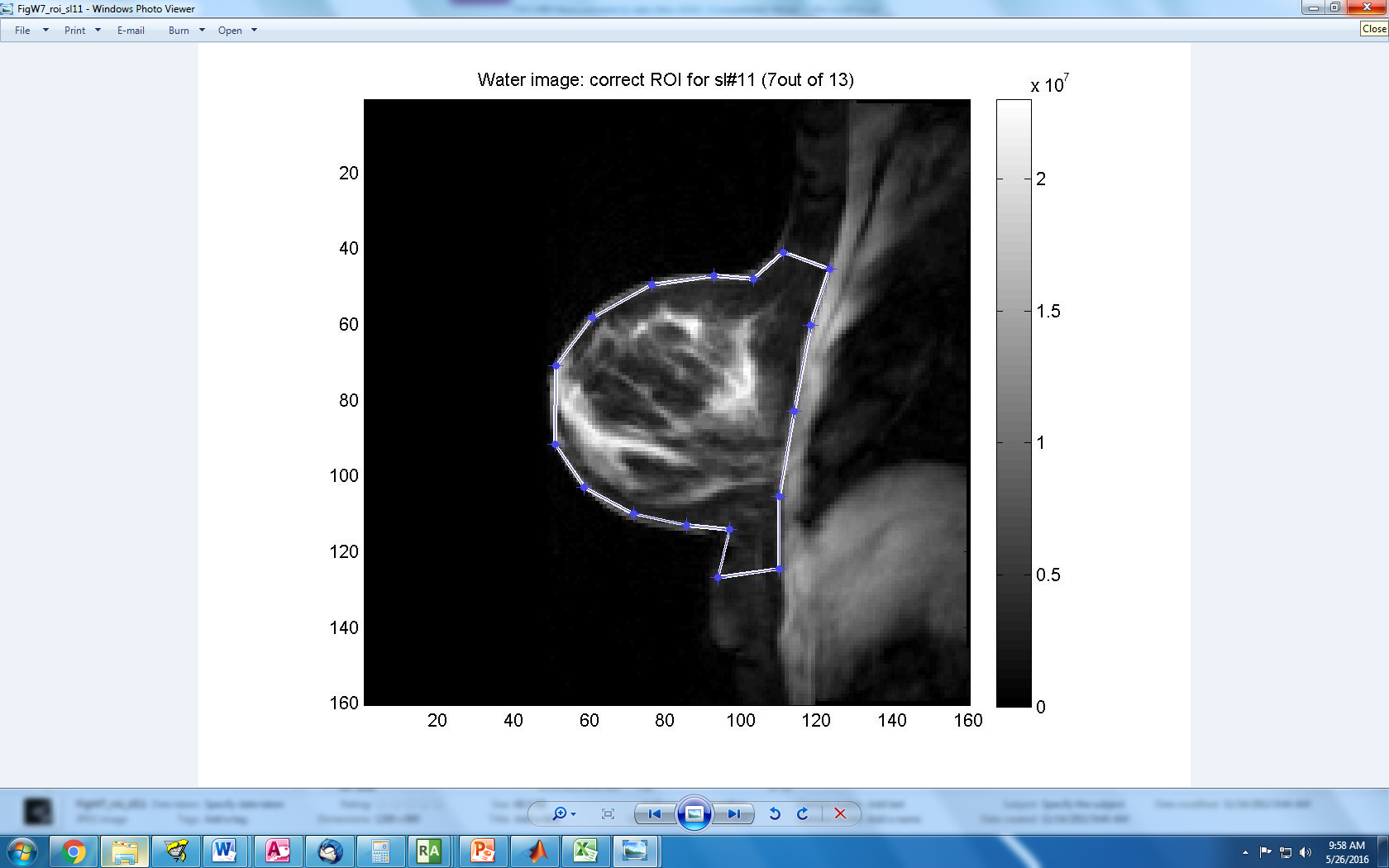

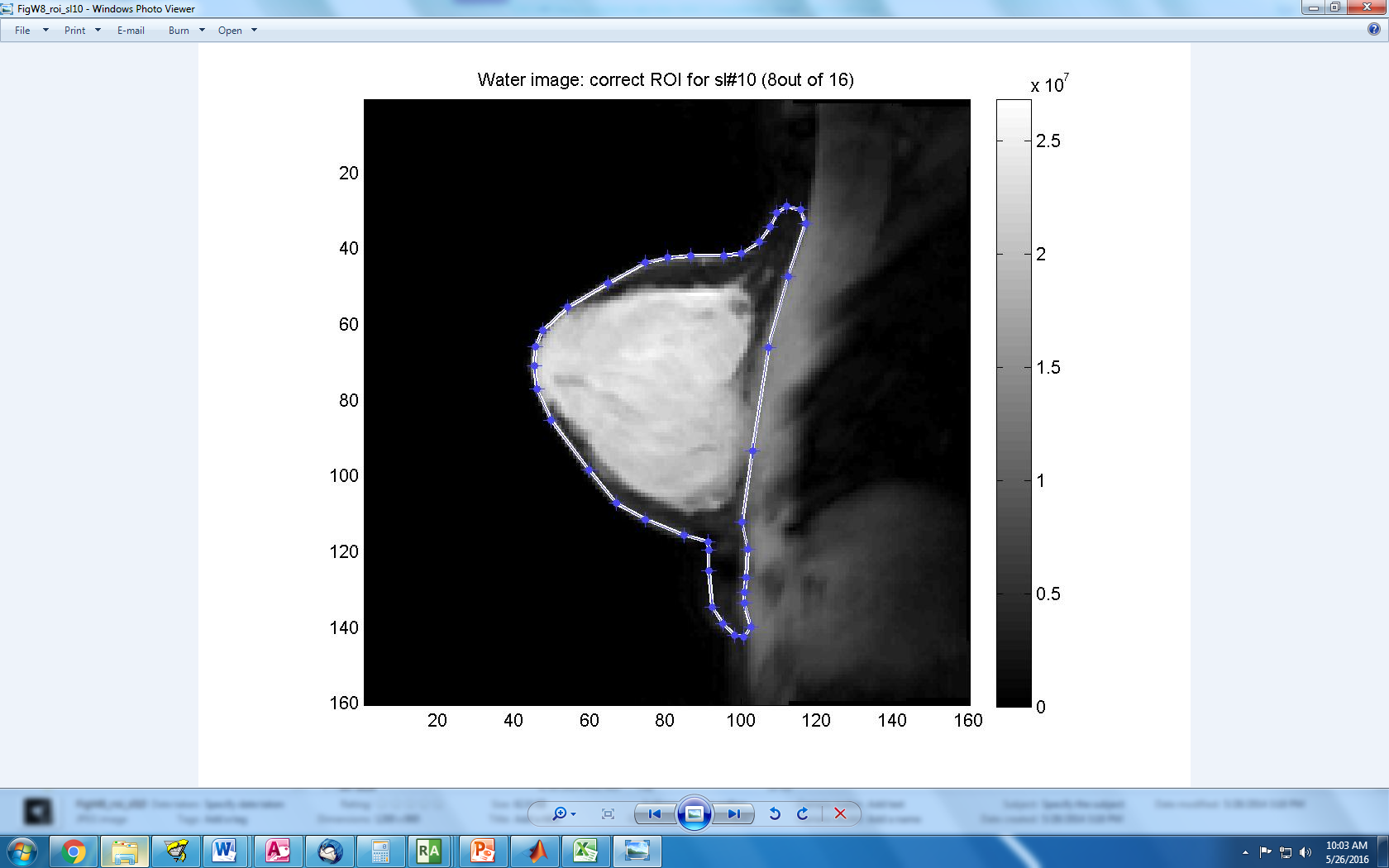

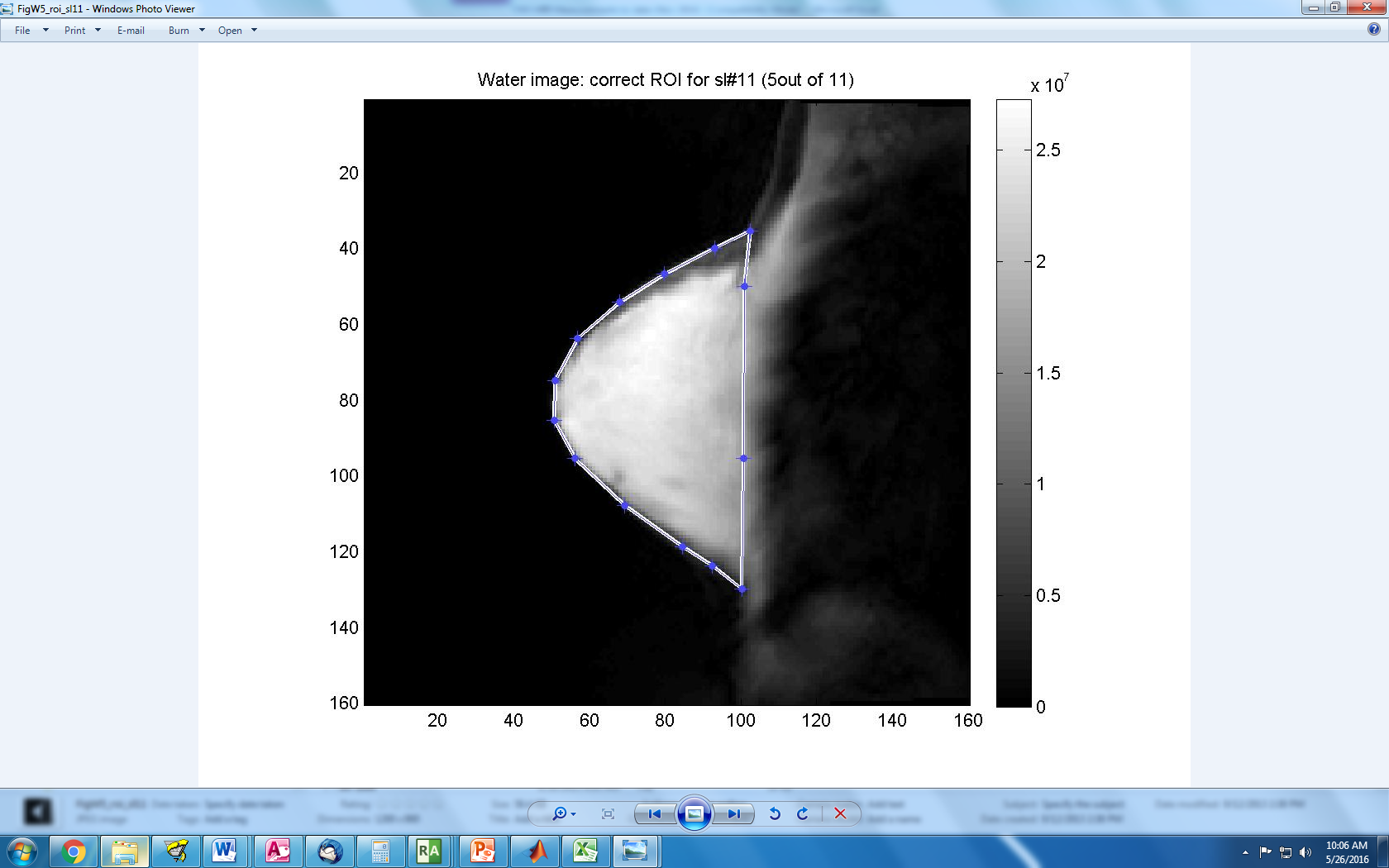


**Supplementary table 1. Associations of age, age at menarche, parity and menopausal status with breast tissue components. Values shown are regression coefficients, adjusted for age, and p values.**

| **Variable** | **Total nuclear area** | **Epithelial nuclear area** | **Non-epithelial nuclear area** | **Collagen** | **Glandular area** |
| --- | --- | --- | --- | --- | --- |
|  | **Estimate (p value)** | **Estimate (p value)** | **Estimate (p value)** | **Estimate (p value)** | **Estimate (p value)** |
| Age | -0.02 (<0.0001) | -0.01(<0.0001) | -0.006 (<0.001) | -0.23 (0.004) | -0.5 (<0.0001) |
| Age at menarche | -0.11 (0.18) | -0.05 (0.34) | -0.06 (0.05) | 1.66 (0.28) | -0.05 (0.71) |
| Number of births | -0.02 (0.50) | -0.02 (0.36) | -0.002 (0.85) | -2.34 (0.005) | -0.05 (0.33) |
| Menopausal status | -0.68 (0.001) | -0.45 (0.001) | -0.23 (0.002) | -10.20 (0.03) | -1.55 (<0.0001) |

Adapted from Table 4 in Li, T. *et al.* The association of measured breast tissue characteristics with mammographic density and

other ris factors for breast cancer. *Cancer Epidemiol Biomarkers Prev* **14**, 343-349, (2005).

**Supplementary Table 2. Selected characteristics of subjects according to study**

| **Variables** | **Study 1A** | **Study 1B** | **Study 2** | **Study 3** | **Study 4** | **Study 5** |
| --- | --- | --- | --- | --- | --- | --- |
| Number of subjects | 1158 | 1128 | 188 | 412 | 1209 | 680 |
| Median age | 16.7 | 49.5 | 52.1 | 53.4 | 56.0 | 57.9 |
| Mean age | 17.9 | 49.4 | 52.6 | 53.9 | 56.7 | 58.9 |
| STD^a^ of age | 3.6 | 4.6 | 8.8 | 9.3 | 9.1 | 11.0 |
| Range of age | 15 - 31 | 34 - 65 | 35 - 77 | 34 – 81 | 37 - 81 | 40 - 85 |
| Ethnicity (% white) | 100 | 100 | 96 | 96 | 97 | 94 |
| Number of subjects with BMI available | 1158 | 1001 | 174 | 410 | 1187 | 679 |
| Mean BMI | 21.5 | 24.4 | 25.6 | 25.7 | 24.9 | 25.5 |
| STD of BMI | 3.4 | 4.3 | 4.5 | 5.2 | 4.3 | 5.3 |
| Range of BMI | 14.5-43.6 | 12.5 – 51.5 | 18.2 – 46.2 | 17.8 – 54.6 | 14.1 – 46.0 | 15.1 – 65.7 |
| Parity (% yes) | 0 | 100 | 96.8  *n = 154* | 80.0  *n=411* | 88.3  *n=1207* | 72.6 |
| Number of live birth among parous women | 0 | 2.3 (0.8)  *n=1003* | 2.5 (0.9)  *n = 149* | 2.7 (1.3)  *n=329* | 3.1 (1.6)  *n=1066* | 2.3 (1.1)  *n=494* |
| Menopausal status  (% post) | 0 | 28.8  *n=1002* | 62.4  *N = 173* | 61.4 | 76.2  *n=1191* | 69.7  *n=679* |
| Median percent density | 49.6^b^ | 31.6 | 32.4 | 35.6 | 23.7 | 27.7 |
| Mean percent density | 48.9 ^b^ | 32.9 | 32.6 | 35.8 | 26.9 | 30.4 |
| Percent density range | 13.2 – 86.5 ^b^ | 0 – 92.6 | 0 - 78 | 0 – 88 | 0 – 81 | 0 – 85 |

Study 1A, Boyd, N. F. et al. Breast-tissue composition and other risk

factors for breast cancer in young women: a cross-sectional study.

Lancet Oncol. 10, 569-580 (2009) and work in progress. (daughters only)

Study 1B Boyd, N. F.et al. Breast-tissue composition and other risk

factors for breast cancer in young women: a cross-sectional study.

Lancet Oncol. 10, 569-580 (2009). (mothers only)

Study 2 Greenwood, C. M. et al. A genome-wide linkage study of

mammographic density, a risk factor for breast cancer.

Breast Cancer Res.13, R132 (2011).

Study 3 Boyd, N. F. et al. Heritability of mammographic density, a

risk factor for breast cancer. N Engl J Med. 347, 886-894 (2002).

Study 4 Boyd N.F. et al. Mammographic density and the risk and

detection of breast cancer. N Engl J Med. 356, 227-236 (2007).

Study 5 Boyd, N. F. et al. Mammographic density and breast cancer

risk: evaluation of a novel method of measuring breast tissue volumes.

Cancer Epidemiol Biomarkers Prev.18, 1754-1762 (2009).

^a^ Standard deviation

^b^ Based on percent water from MRI measures

**Supplementary Table 3. Comparison of observed and predicted age-specific breast cancer incidence using three predictive models (including young woman with calibrated percent water).**

| **Age** | **Breast Cancer Incidence per 100,000 (O)** | **Predicted Breast Cancer Incidence - log (age) (P^1^)** | **Ratio O/P^1^** | **Predicted Breast Cancer Incidence – log (CBD) (P^2^)** | **Ratio O/P**^2^ | **Predicted Breast Cancer Incidence – log (CBD) and log (age) (P^3^)** | **Ratio O/P^3^** |
| --- | --- | --- | --- | --- | --- | --- | --- |
|  |  |  |  |  |  |  |  |
| **15-19** | 0.3 | 0.76 | 0.39 | 0.21 | 1.43 | 0.19 | 1.58 |
| **20-24** | 1.2 | 2.42 | 0.49 | 2.06 | 0.58 | 2.07 | 0.58 |
| **25-29** | 6.4 | 6.09 | 1.05 | 8.22 | 0.78 | 8.58 | 0.74 |
| **30-34** | 21.7 | 13.14 | 1.65 | 21.86 | 0.99 | 23.18 | 0.94 |
| **35-39** | 52.4 | 25.39 | 2.06 | 46.74 | 1.12 | 49.82 | 1.05 |
| **40-44** | 105.1 | 45.17 | 2.33 | 81.46 | 1.29 | 86.34 | 1.22 |
| **45-49** | 162 | 75.35 | 2.15 | 127.18 | 1.27 | 133.49 | 1.21 |
| **50-54** | 207.2 | 119.43 | 1.73 | 172.48 | 1.20 | 177.85 | 1.16 |
| **55-59** | 252.5 | 181.50 | 1.39 | 218.58 | 1.15 | 220.92 | 1.14 |
| **60-64** | 306 | 266.38 | 1.15 | 271.05 | 1.13 | 268.92 | 1.14 |
| **65-69** | 335.2 | 379.58 | 0.88 | 321.24 | 1.04 | 312.44 | 1.07 |
| **70-74** | 342.5 | 527.35 | 0.65 | 375.51 | 0.91 | 358.51 | 0.95 |
| **75-79** | 355.8 | 716.74 | 0.49 | 435.24 | 0.82 | 408.46 | 0.87 |
| **80+** | 346.6 | 955.64 | 0.36 | 489.92 | 0.71 | 451.57 | 0.77 |
|  |  |  |  |  |  |  |  |
| **R^2^** |  | 0.88 |  | 0.99 |  | 0.99 |  |

O=observed, P = predicted by models 1, 2, or 3; CBD=cumulative breast density.

Models:

1. Log (breast cancer incidence) = -13.44+ 4.6 log (age).

2. Log (breast cancer incidence) = - 21.17 + 3.5 log (calibrated cumulative breast density).

3. Log (breast cancer incidence) = -21.8+ 3.87 log (calibrated cumulative breast density) – 0.47 log (age).
